# Bioerosion by pit-forming, temperate-reef sea urchins: History, rates and broader implications

**DOI:** 10.1371/journal.pone.0191278

**Published:** 2018-02-21

**Authors:** Michael P. Russell, Victoria K. Gibbs, Emily Duwan

**Affiliations:** Biology Department, Villanova University, Villanova, Pennsylvania, United States of America; Department of Agriculture and Water Resources, AUSTRALIA

## Abstract

Sea urchins are dominant members of rocky temperate reefs around the world. They often occur in cavities within the rock, and fit so tightly, it is natural to assume they sculpted these “pits.” However, there are no experimental data demonstrating they bore pits. If they do, what are the rates and consequences of bioerosion to nearshore systems? We sampled purple sea urchins, *Strongylocentrotus purpuratus*, from sites with four rock types, three sedimentary (two sandstones and one mudstone) and one metamorphic (granite). A year-long experiment showed urchins excavated depressions on sedimentary rocks in just months. The rate of pit formation varied with rock type and ranged from <5 yr for medium-grain sandstone to >100 yr for granite. In the field, there were differences in pit size and shapes of the urchins (height:diameter ratio). The pits were shallow and urchins flatter at the granite site, and the pits were deeper and urchins taller at the sedimentary sites. Although overall pit sizes were larger on mudstone than on sandstone, urchin size accounted for this difference. A second, short-term experiment, showed the primary mechanism for bioerosion was ingestion of the substratum. This experiment eliminated potential confounding factors of the year-long experiment and yielded higher bioerosion rates. Given the high densities of urchins, large amounts of rock can be converted to sediment over short time periods. Urchins on sandstone can excavate as much as 11.4 kg m^-2^ yr^-1^. On a broader geographic scale, sediment production can exceed 100 t ha^-1^ yr^-1^, and across their range, their combined bioerosion is comparable to the sediment load of many rivers. The phase shift between urchin barrens and kelp bed habitats in the North Pacific is controlled by the trophic cascade of sea otters. By limiting urchin populations, these apex predators also may indirectly control a substantial component of coastal rates of bioerosion.

## Introduction

**Fig 1:**
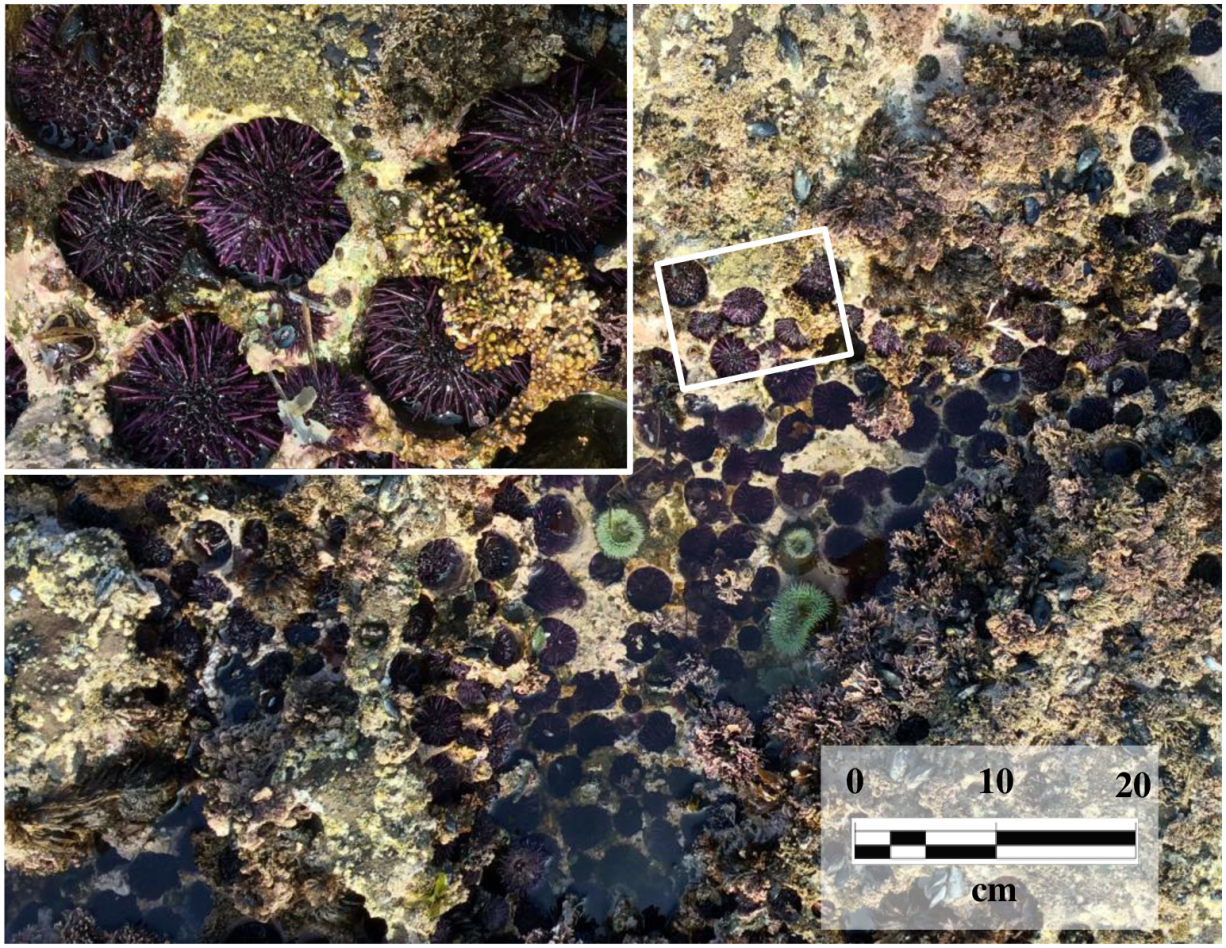
Rocky intertidal pool. Purple sea urchins occur in high densities in the intertidal and shallow subtidal where many are nestled in cavities (“pits”) carved out of the rock substratum. The “hand-in-glove” fit of the urchins to the pits is apparent upon close examination (inset). The sedimentary sandstone at this site (Bean Hollow, California, USA) is typical of many sites along the west coast of North America. The exposed rocks at this site are of the Upper Cretaceous Pigeon Point Formation, which is part of the Franciscan Complex that makes up much of the central coast of California.

The high densities and intense grazing of sea urchins drive the composition of many nearshore communities [1–5]. One of the most striking characteristics of temperate and subtropical rocky reefs is the cavities in the substratum that sea urchins occupy (Fig 1). The fit is so close they appear to have sculpted these pits. Nineteenth century naturalists observed this association [6], speculated about its origin, and debated whether urchins were excavating pits or the pits resulted from weathering and abiotic erosion processes [7–10]. Proponents of the active boring hypothesis speculated about the specific mechanism(s) of pit formation suggesting the grasping by tubefeet [11] or scraping by the teeth of the feeding structure, Aristotle’s Lantern [8, 12]. The magnesium-enriched calcite of urchin teeth resembles composite materials imparting considerable strength as they rasp across hard surfaces [13]. Temperate-reef sea urchins occur in pits on different types of metamorphic and sedimentary rock reef, e.g., granite and sandstone [14]. Although later workers assumed sea urchins were responsible for the pits, they did not know the rate of formation [15]. Today the question remains, if temperate sea urchins are boring pits, what are the rates and consequences of their bioerosion?

Despite their abundance, ubiquity in these systems, and the obvious close association they have with pits, there are no studies quantifying the rate of pit formation or the implications of the resulting geomorphology and erosion processes. Studies that claim sea urchins actively bore pits primarily cite two references [16, 17]. However, while both literature reviews make well-reasoned and strong cases, they provide no data or experimental evidence that demonstrate sea urchins excavate rocky substrata. In their book, “Between Pacific Tides”, Ricketts and Calvin [18] write about *S*. *purpuratus* that Fewkes [17] “stated the situation correctly in 1890” that abrasion from teeth and spines “account for the pits.” Other than the obvious close fit of the occupant to the cavity (Fig 1), the only evidence indicating sea urchins possess the ability to bore into rock were observations in the 1800s that the teeth of excised Aristotle’s Lantern could be used to successfully gouge small depressions [19, 20]. If one accepts the close-fitting association of urchins with their pits as evidence of their ability to excavate rock, there are still no quantitative estimates of how long it takes. In 1911, Romanes [15] questioned their ability to bore rock and asked “Whether the action is a fairly rapid one … or … the result of generation after generation”. After more than one century these questions remain.

Here we test the hypothesis that sea urchins actively bore pits, and quantify the rate and method of pit excavation. Urchin pits are just part of the complex topography of rocky shores (Fig 1), so to isolate the effects of urchins, we used individuals on standardized (flat), rocky substrata, and showed they are capable of excavating pits. The changes in mass of these substrata were used to quantify rates of bioerosion and pit formation in the field. These rates were calculated on a per urchin basis and extrapolated to population-level, and larger geographic-scale, bioerosion and sediment production estimates. Historically, changes in sea otter populations along the range of *S*. *purpuratus* have resulted in the alternative stable states of urchin barrens and kelp beds. We speculate that this dynamic indirectly controls the level of bioerosion produced by urchins in this trophic cascade.

## Materials and methods

### Ethic statement

Samples in this study were obtained in compliance with all federal and state regulations under the Scientific Collecting (SC) permit issued by the Department of Fish and Wildlife (California Natural Resource Agency) #SC-9365 to M. P. Russell. Fields sites are public access and *S*. *purpuratus* is not an endangered or protected species.

### One-year experiment

**Fig 2:**
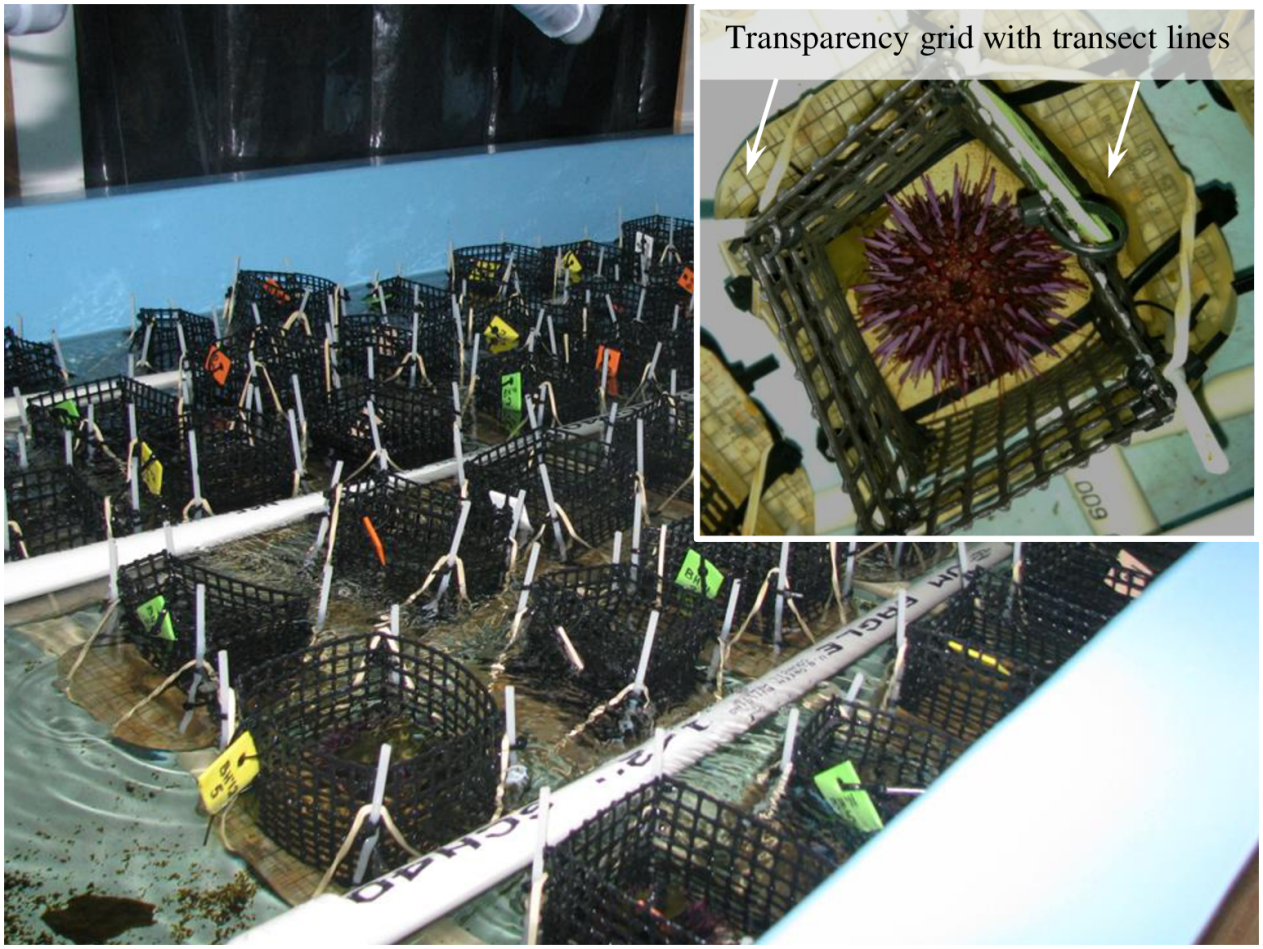
Oblique view of the sea table and experimental layout. Each experimental unit was secured to a PVC-grid on the bottom of the table (not visible, covered by units); the 3 pipes of the sprinkler-system that delivered filtered seawater are visible. For each replicate, the plastic cages surrounding the rocks were secured to the epoxy units with cable ties and plastic posts. The cages restricted urchin movement to the rock surfaces. An overhead view of a single replicate (inset) shows an urchin on one of the sandstone (fine-grain) surfaces and the standardized grid lines for the perpendicular transects used to quantify surface topography. We used a carpenter’s contour gauge along these transects to measure the height of the rock (every 0.5 cm) above the epoxy base. These topographic data were used to generate three-dimensional surface plots and calculate rugosity at the start and conclusion of the experiment.

Over the past three decades [21], we periodically sampled dozens of intertidal *S*. *purpuratus* sites throughout its geographic range (Torch Bay, Alaska, USA [58° 19’ N, 136° 48’ W] to Los Ojitos, Baja California del Norte, Mexico [28° 54.5’ N, 114° 27’ W]) [22] and observed sea urchins in pits on a variety of metamorphic and sedimentary rock substrates. We selected three sites for source substrates that represented the variety of rock types: mudstone, sandstone, and granite. In a laboratory experiment, four treatments were established, one each from granite and mudstone, and two from sandstone (fine- and medium-grain size). We used these rocks to create individual units (replicates) that consisted of a flattened section of rock (~ 6 cm x 6 cm) and placed a sea urchin (see below) on each one for one year (Fig 2). Control replicates had rocks but no sea urchins. We quantified density of the four rock types, and at the start, 6-months, and one year later, quantified: weight of the rock units, surface topography (rugosity), and sea urchin size. At the conclusion of the experiment we dissected all the sea urchins and recorded dry weights of the different body components: Aristotle’s Lantern, test and spines, gonad, digestive tract (gut), and gut contents. The experiment began on August 29, 2012 and concluded on August 27, 2013.

### Source of rock substrata

We collected four types of rock from three sites in California: granite at Bodega Bay (38° 19ʹ 08.28ʺ N 123° 04ʹ 27.85ʺ W); mudstone at Palomarin Beach (37° 55ʹ 48.81ʺ N 122° 44ʹ 44.09ʺ W); and two types of sandstone from Bean Hollow (37° 13ʹ 36.08ʺ N 122° 24ʹ 41.70ʺ W). The granite is a Cretaceous hornblende-biotite quartz diorite and is part of the Salinian block [23]. The organic-rich siliceous mudstone from Palomarin is olive gray-green to yellow-brown and part of the Upper Miocene Santa Cruz Formation [24]. The sedimentary rocks at Bean Hollow are of the Upper Cretaceous Pigeon Point Formation, itself a part of the Franciscan Complex that makes up much of the Central Coast of California [25]. The grain lithology within both sandstones is approximately 50% quartz, 35% feldspar and 15% lithic fragments that include felsic volcanic rocks, plus minor amounts mica and other minerals. The grain shapes range from sub-angular to sub-rounded and the grains are weakly to moderately cemented by amorphous silica. The difference in the two sandstones (collected from different exposed beds) was grain size—medium (moderately well-sorted, course to medium sand, 1.00 to 0.25 mm diameter) and fine (well sorted, fine to very fine, 0.25 to 0.063 mm).

### Experimental units

Replicates were constructed from the different rock substrates, which were cut or ground to expose a flat surface to individual sea urchins. Rectangular sandstone and granite pieces were cut using a wet masonry saw with a 14″ diamond blade. The specific dimensions of the pieces varied with the size and shape of the original rock samples. The mudstone samples splintered and shattered under the diamond blade making it impossible to use the masonry saw on this rock type. Instead, we collected flattened cobbles derived from the mudstone bedrock at the Palomarin site and ground an even plane into these natural units on the side of greatest surface area using a disc sander, first with course (50) and then fine (100) grit (see below for potential effects of disc-grinding).

The cut/sanded rock units from the four different substrates (n = 10 for each) were placed into the center of a square (10 cm x 10 cm) layer of marine epoxy (1.5–3.0 cm depth, West System^®^ 105) so that 0.5–1.0 cm of the flattened, exposed surface of the rock, was above the epoxy (Fig 2). After the epoxy hardened, each replicate was measured for a customized 10x10 cm grid (0.5 cm increments) printed on a transparency sheet. Each transparency grid had the footprint of the rock cut out and then was epoxied around the exposed rock surface (Fig 2 inset). This grid provided standardized Cartesian coordinates to measure the height of the rock units (topographic relief and rugosity) relative to the surface of the epoxy. On the opposite side from the exposed rock, a small (1.5–3.0 cm) length of PVC pipe (diameter = 1.27 cm) was epoxied to the center to serve as a “post”. To constrain the movement of the sea urchins to the rock surfaces, four-sided (open-top), plastic mesh-fences (7.25 cm tall; diagonal opening of mesh = 0.9 cm) were used. The four corners of the fence had plastic posts fitted into drilled holes in the surface of the epoxy. The posts and bases of the fence were secured to the units with elastic bands and cable ties. In addition to the replicates that had sea urchins we had two types of controls: replicates with rocks and no sea urchins (n = 3 each for granite, mudstone, and the medium-grain sandstone only) and replicates with neither rocks nor sea urchins (n = 5 of epoxy-only replicates).

We randomly assigned the 54 units to a position in a 9 x 6 grid constructed of 1.27 cm PVC pipe. The grid sat on the bottom of a sea table (183 x 92 cm x 30 cm deep) that was part of a filtered, 1000-L recirculating seawater system (Fig 2). The posts on the bottom of the units fit into PVC T’s in the grid. Filtered seawater was delivered to each unit via a PVC sprinkler system (a series of three 1.27 cm PVC pipes separating the adjacent 6 rows of units—each sprinkler had 9 paired 0.3 cm holes that delivered a jet of water through the side of the plastic fence). A standpipe maintained the depth of water in the sea table so that the tops of the fences of the units were above the water line (water depth ~ 18 cm) to keep the urchins confined to the rock units. Water pressure varied along the lengths of the sprinkler pipes so the positions of the units were systematically rotated in the grid every day (Fig 2).

### Sea urchin collection, maintenance and feeding

We collected samples of intertidal purple sea urchins, *S*. *purpuratus*, on July 2, 2012 at Bean Hollow and shipped them to our lab (Villanova University). We kept the urchins in the sea table and maintained the water at 12.5°C (±0.49), and salinity = 31.5‰ (±0.69) [these and all subsequent ± values are standard deviations (sd)]. For the one-year experiment, we size-sorted the urchins and selected 40 that fit within a limited range (29.93–36.10 mm test diameter). These were further sorted into four groups such that the test diameter means and sd (mm) of the groups were as equal as possible: 33.41±2.14, 33.21±1.98, 33.14±1.63, and 32.94±1.53. These groups were randomly assigned to the four rock treatments. The remaining urchin samples (~50) were housed in two open-mesh cages in the sea table. Each day, the standpipe in the sea table was removed and the units and all surfaces thoroughly flushed with filtered seawater. The drain below the table was fitted with a 100 μm filter to catch fecal pellets which were discarded from the system.

We used two types of food; initially, fresh-frozen kelp, and later, a commercial dried kelp. For the first six months, the urchins on the experimental units fed on kelp we had shipped from near Bean Hollow. We cut kelp blades into strips (~1.6–2.5 cm^2^), froze them in seawater, defrosted before feeding, and provided them to each unit (including the two types of controls without urchins). Kelp strips were not weighed but were provided in approximately equal amounts (1–2 strips d^-1^). The cost and handling time of this kelp forced us to switch to the commercial dried kelp for the last six months. We fed the extra urchins housed in the open-mesh cages the fresh-frozen kelp for the first two months, but then switched to re-hydrated dried kelp. When we first started using the dried kelp, we simply rehydrated it overnight in a beaker (see below).

After using the commercial dried kelp (feeding the extra urchins) in the seawater system for 8 weeks, we discovered several urchins on both the rock units, and cages with extra urchins, with broken teeth. Fragments of teeth (ranging in size from distal tips to approximately 75% of the tooth length) were found in the sea table during the daily cleaning and removal of fecal material. Dissections (n = 10) of a subsample of the extra urchins revealed 9 with various stages of broken/missing teeth. We traced the problem to lowered Ca and Mg levels in the seawater, which were caused by increased levels of PO_4_^-3^ from the rehydrated commercial kelp. Sea urchin teeth are composed of Mg-rich crystalline calcite and are self-sharpening [26]. The teeth grow continuously and are capable of regenerating the entire length approximately every 100 days [27]. The reduced levels of Ca and Mg compromised the structural integrity of the teeth. Over the next 8-week period, we balanced the water chemistry levels by supplementing the filtered seawater with Ca and Mg and incorporating a PO_4_^-3^ chelator (Phosban ^®^) as part of the filtration system. We eliminated the additional PO_4_^-3^ from the rehydrated commercial kelp by thoroughly rinsing it with tap and deionized water over a 5-day period. By the sixth month of the experiment, no further tooth breakage was noted, and all sea urchins regenerated the full complement of their teeth.

### Lab measurements

We recorded the dry weights of all units (the rock embedded in epoxy with PVC standpipe, not the plastic cages, elastic bands, or cable ties) two times. First, before placing sea urchins on them, and then after one year of grazing. Dry weights were also recorded for the two types of controls—replicates with rocks and no urchins, and epoxy-only units with neither rocks nor urchins. Before drying, all units were rinsed (not scrubbed) in deionized water to remove any salt. After one year in the seawater system, the epoxy-only units gained an average of 1.29 ±.03 g. We attribute this gain to biofilm growth on the surface—although there was no visible scum coating the surface, the units were slimy to the touch after being submerged in the seawater over the course of the year. After the orbital-sanding process, one of the mudstone control units splintered during the first week of the experiment and had to be eliminated. To isolate the effect of sea urchins on the change in dry weight (bioerosion estimate) and eliminate either loss of weight from seawater weathering/dissolution, or weight gain from build-up of a biofilm, the dry weight of units with urchins was adjusted using the mean dry weight change of control units that had rocks but no sea urchins. The medium-grain sandstone treatment visibly lost more material than the fine-grain. Although we did not have fine-grain controls, we used the mean percent control adjustment of all medium-grain treatments for each fine-grain sandstone unit.

To quantify the change in topography, we used a carpenter’s contour gauge, and the transparency grid on each unit provided permanent, orthogonal transects-lines, every 5 mm (see inset on Fig 2). Surface contours were standardized to the level of the epoxy across all units. The elevation (± 0.25mm) of the rock above the surface of the epoxy was recorded on the two sets of perpendicular transects so the height at each intersecting point of the grid was measured twice. We used the mean of these values and Delaunay triangulation (DelaunayTri [28]) to reconstruct the 3-D surface areas. From these reconstructions, we calculated real surface area (A_r_) and geometric (projected) surface area (A_g_), which yielded estimates of rugosity (A_r_/A_g_).

The change in dry weight of the rock units was used to estimate the volume that the urchins bioeroded. To convert weight to volume, we first estimated the density of different rock samples of the four substrate types (n = 10 each) by calculating the volume displaced. Because the rocks are porous, we measured the displacement of sand, not water. Fine sand was dried and sieved multiple times to produce a matrix of uniform density and particle size (n = 10, 1.6421 g ml^-1^ ± 0.0089). The dry weights of each rock sample were recorded and placed in a beaker. We filled the beaker with the sand matrix to a known volume and then recorded the weight of the sand matrix, which was used to calculate its volume. The difference between the known volume of the contents of the beaker and volume of sand was the volume of the rock. These volume and weight estimates of the rock yielded estimates of rock density.

At the conclusion of the laboratory experiment, all urchins were dissected into body components: Aristotle’s lantern, body (test and spines), gonad, digestive tract (gut), and gut contents. Body components were dried at 50°C for one week before recording weights.

### Field measurements

In the field we measured pit depth and diameter on the different rock types and the size (test height and diameter) of the urchin occupants. We quantified pit volume at each site by identifying urchin pits and averaging two measures each of diameter ($\bar{d}$ –the two measures were orthogonal) and depth ($\bar{h}$ –at the center). The volume (*V*) of each pit was estimated using a cylinder:

$$V=\pi {\left(\frac{\bar{d}}{2}\right)}^{2}\bar{h}$$ (1)

At two of the sites (Palomarin—mudstone, and Bean Hollow—sandstone), we also measured the height and diameter of the urchins occupying the pits (July 23–25, 2013). At the granite site (Bodega Bay) there was a massive die-off, and purple sea urchins were “functionally extirpated” in August of 2011 [29]. The suspected cause was a localized harmful algal bloom. Because of the die-off at Bodega Bay, we could only measure pits at this site (July 22, 2013); there were no occupants. However, this rock type occurs at another site (Montara Lighthouse) 90 km south, outside the affected mass-mortality area [29] where we quantified both urchin and pit size in a tidepool (37° 32ʹ 14.46ʺ N 122° 31ʹ 11.13ʺ W) on July 13, 2014. The pit volumes from Bodega were not significantly different (χ^2^ = 0.065, *p* = 0.80).

### Waste collection experiment

Three dissections from the one-year bioerosion experiment revealed boluses in the digestive tracts that were composed of sedimentary particles of the medium-grain sandstone treatment (S1 Fig). To further quantify the ingestion of these particles as part of the mechanics of the bioerosion process, we followed the one-year experiment with a short-term (7 weeks) waste-collection experiment. We used the same rock replicates, but with three changes. Instead of laying them out on a grid in the sea table, each unit was placed in an individual 4.7 L plastic bucket, which collected all the waste (fecal pellets and other material–S1 Fig). Second, the surfaces of the rock units were scraped with a serrated knife to expose a “clean” layer (see Discussion below concerning possible effects of disc sanding the mudstone units). Third, the control units for this experiment were smooth glass tiles (10.7 x 10.7 cm) that the urchins could not ingest or erode. We measured the dry and ash (i.e., inorganic content) weights of the waste (from weeks 2 through 6) and gut contents (at the end of the experiment). The controls provided the background levels (inorganic/organic percentages of the waste and gut contents from the kelp diet) without any contribution from bioerosion. These glass control units were constructed in the same way as the rock units (with cages). Space constraints limited replicates to n = 5 each for the 5 treatments: control, fine-grain sandstone, medium-grain sandstone, mudstone, and granite.

The urchins for this experiment were collected from the same site (Bean Hollow) on July 24, 2013, size sorted into groups, and the groups randomly assigned to the five treatments. The mean test diameters (mm) of the groups were: 36.52±1.95, 36.53±2.22, 37.44±1.61, 37.71±2.02, and 38.63±1.67. We fed each urchin 1.05 ± .02 g of rehydrated (and thoroughly rinsed) kelp every day (equal to 5% of the average weight of all urchins). The seawater was maintained at constant temperature (12 ± 1.5°C) and salinity (32 ± 1 ppt). The flow rate to each unit was approximately 1 L•min^-1^ and the positions of the buckets rotated in the sea table every other day to account for any variation in flow rates.

We discarded the waste material from the buckets for the first week to insure this material was the result of grazing on the rock units. Then every other day for the next 5 weeks, all the waste material was collected from the buckets (the waste material was not processed during the 7^th^ week). After removing the urchins from the units, we filtered the contents of each bucket (excluding any broken spines tips) through a 100 μm sieve.

This filtered material, or “waste residue”, was primarily fecal pellets and some particles from the rock units, and was rinsed in deionized water to remove any salt. At the end of each of the five weeks we recorded the dry and ash weights of the waste residue (see below) and summed these values for the entire experiment. As in the one-year bioerosion experiment, at the conclusion we dissected all the sea urchins and recorded dry weights of the different body components. In addition, we also measured ash weights of the gut contents.

To distinguish the amount of waste material attributable to ingested algae expelled as fecal material, from bioerosion activities of the urchins, we measured the organic and inorganic fractions (via dry and ash weights) of the waste residue (throughout the experiment) and the gut contents from the dissections at the end of the experiment. In addition, we measured the dry weight of the material lost from each unit (including the controls) and the inorganic/organic fractions of the four rock types.

All samples were dried at 50°C (minimum 72 hours) to a constant weight before recording dry weights. Ash weights were obtained by burning off the organic component in a muffle furnace for 4 hours at 500°C. This method was also used for samples of the 4 rock types (n = 7 for each type) to determine their organic content. Samples were rinsed in deionized water, air dried, and pulverized with a mortar and pestle before processing for dry and ash weights.

The waste residue from the smooth glass controls provided the inorganic percentage of fecal pellets alone because there were no sedimentary particles in this waste residue. We used this percentage to calculate the amount of the inorganic waste residue from the rock units in the other treatments. Similarly, the controls provided the inorganic percentage of gut contents from ingested algae alone, and we used this percentage to calculate the amount of the inorganic gut content from the rock units in the other treatments. For all the replicates of the four rock treatments, we regressed these values (inorganic waste residue not from fecal material, inorganic gut content not from algae ingested) as a function of the change in the inorganic weight of the experimental units.

At the end of the experiment we observed that the inorganic material lost from the rock units was much greater than the inorganic material recovered from the waste residue plus the gut content (see Results). We suspected the difference was due to fine particles washing through the 100-micron sieve with the filtrate. We calculated the amount of inorganic material lost from the rock blocks that is not accounted for (% Lost) in the inorganic residue plus inorganic gut content:

%Lost=((ΔWeightRockBlock)–(Residue+GutContent))/(ΔWeightRockBlock) (2)

For sedimentary rocks we tested if grain size was associated with % Lost and predicted that mudstone (the smallest grain size) should show the highest % Lost and medium-grain sandstone (the largest grain size) the least.

### Comparison of lab experiments

A direct comparison between the bioerosion rate estimates on the different rock blocks between the one-year, and waste collection experiments, was possible because we used the same units in both. We prorated the change in dry weight of the waste collection units to one year (multiply by 52/7 –the one-year was 52 weeks and the waste collection lasted 7 weeks). Each rock unit (n = 5 for each treatment) yielded two estimates and we plotted these against each other.

### Statistics

All analyses were carried out using *R* [30]. For comparing 2 or more groups we used ANOVA if assumptions were satisfied, and Kruskal-Wallis test if not. We evaluated the assumption of a normal distribution on the residuals using Shapiro-Wilk test and the assumption of homoscedasticity using Bartlett’s test. We used a one-tail Wilcoxon sign rank test with continuity correction for analyzing change in elevation (Δ elevation = 0) for comparing the experimental units in the one-year bioerosion experiment. A Principle Component Analysis (PCA) of the one-year experiment dry-weight dissection data yielded a PC that accounted for 57.7% of the total variance; we used ANOVA on this component to compare treatment groups. Overall growth rates for the year were analyzed with ANOVA. To compare growth during the first versus last six months we used the nonparametric Friedman’s test because variance was heteroscedastic. We compared urchin pits in the field with an ANCOVA on cube-root of pit-volume (ml^1/3^), with cube-root of test volume of the urchin-occupant as the covariate because of the differences among sites in urchin sizes. Finally, we used Model II regressions for the waste-collection experiment and for comparing bioerosion rates between the year-long and waste-collection experiments.

## Results

### One-year experiment

**Fig 3:**
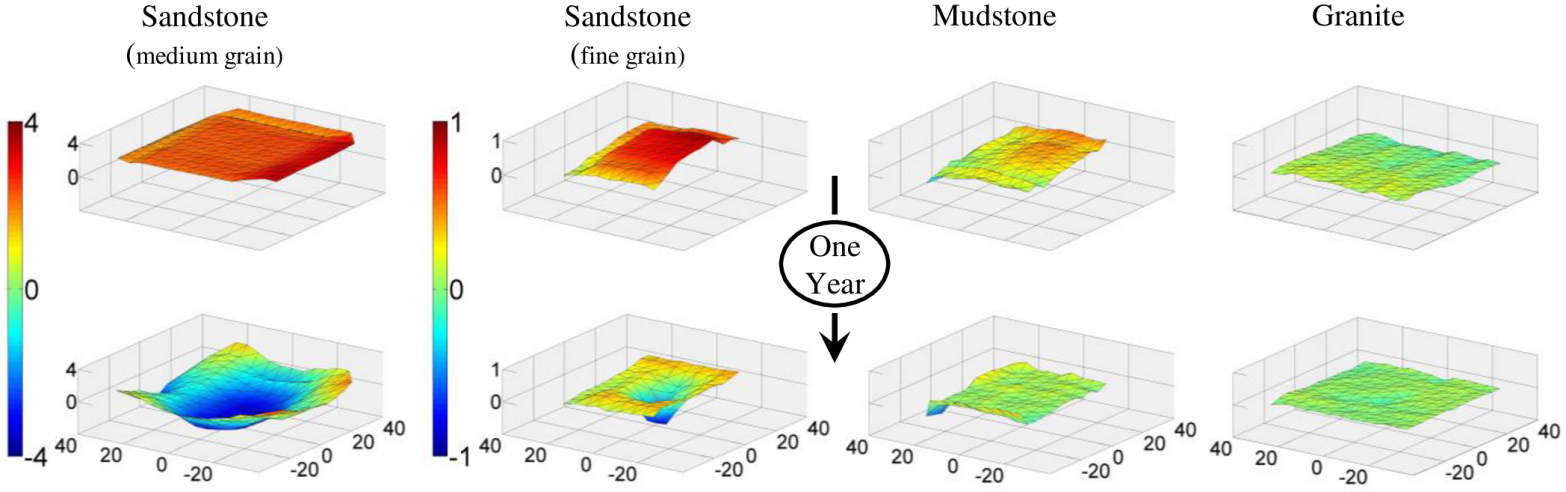
Three dimensional surface plots. Rock substrates used in the one-year experiment. Units on all axes are mm and plots are means (n = 10) at the start (top) and after the exposure of each replicate to a single grazing sea urchin for one year (bottom). The medium-grain sandstone showed the most intense levels of bioerosion and pit formation and the relief (color) z-axis scale is 4x the scale for the fine-grain sandstone, mudstone, and granite.

There was a clear difference among treatments in pit formation over the year (Fig 3) [28]. There were significant differences in the change in rugosity between treatment groups (χ^2^ = 25.3, *P* < .0001), except between mudstone and granite (*t* = 0.158, *P* = 0.85). All the medium-grain sandstone replicates showed obvious signs of pit formation, while the fine-grain sandstone replicates showed moderate to low levels of pit formation. None of the granite replicates showed obvious signs of pit formation whereas 3 mudstone units showed initial signs of pit formation, and one had an obvious pit. All treatments showed an overall reduction in weight (Table 1) and elevation (granite, V = 4992, *P*<0.01; mudstone, V = 291, *P*<0.0001; fine-grain sandstone, V = 0, *P*<0.0001; medium-grain sandstone, V = 61, *P*<0.0001) indicating that some bioerosion occurred even in the least friable substrates.

**Table 1:** Rock types, density, pit volume, and bioerosion estimates, ± is standard deviation.

Rock ^a^	Density^a^	Lab bioerosion^a^	Field Pit size^b^	Rate^c^	Field bioerosion^d^
	(g cm^-3^)	(g yr^-1^)	(cm^3^)	(yr)	(t yr^-1^ ha^-1^)
Granite	2.85 ± 0.28	0.88 ± 0.35	45.5 ± 32.1	147.5	2.9
Mudstone	1.77 ± 0.17	2.73 ± 2.84	220.3 ± 79.2	143.2	4.2
Sandstone (med)	2.30 ± 0.25	32.40 ± 8.14	63.5 ± 34.4	4.5	199.1
Sandstone (fine)	2.40 ± 0.46	4.78 ± 2.12	63.5 ± 34.4	31.9	29.4

^a^ Means (n = 10) for each rock type.

^b^ Sample sizes varied: Granite (Bodega Bay and Montara Lighthouse, n = 23), Mudstone (Palomarin, n = 47), and Sandstone (same for both med = medium-grain, fine = fine-grain, Bean Hollow n = 49).

^c^ Time if a single urchin were to make a site-specific average size pit based on pit size in field and lab bioerosion.

^d^ Based on lab estimates of bioerosion and site-specific urchin density [S1 Table].

We used the mean weight changes in each of the four types of rock units to estimate the rates of bioerosion and pit formation (Table 1). In the field we sampled urchins in pits and measured both the test height and diameter to calculate urchin volume [31]. The size of the pits they occupied was estimated by calculating the volume of a cylinder from the depth and diameter of the pit. Based on the densities of the different rock types, we calculated the mean excavation rates (cm^3^ yr^-1^). From these estimates we calculated the time it would take if a single urchin were to form an average-size pit on the different substrates (but see below for caveats on urchin sizes, behavior, and pits in the field). These excavation rates ranged from <5 years on medium-grain sandstone to >100 years on mudstone and granite (Table 1). Using site-specific counts of urchins in tidepools (S1 Table) we estimated urchin density and larger scale field bioerosion rates in t yr^-1^ ha^-1^ (Table 1).

**Fig 4:**
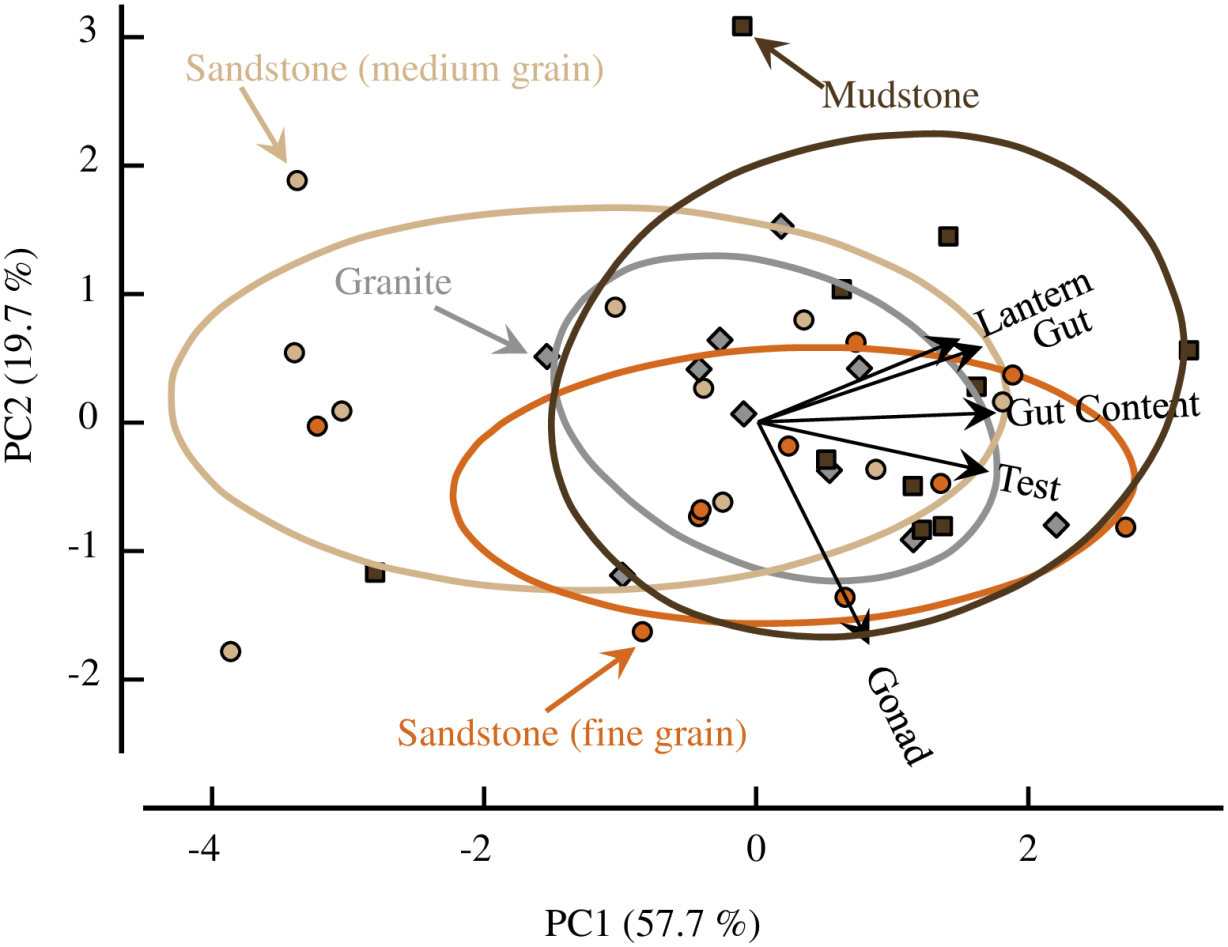
Principal component analysis. The first two principal components accounted for 77.4% of the variation. The four treatments (n = 10) are distinguished by different colored symbols which match the colors of the 68% confidence ellipses for each group: sandstone = circles (tan = medium-grain, orange = fine-grain); mudstone = brown squares; granite = gray diamonds. Vectors of loadings for the five different body components are also plotted (black arrows).

The PCA of the dissection data revealed no striking differences among the four treatments (Fig 4). Although the ANOVA of PCA 1 showed a significant effect of treatment (F_3,36_, p = 0.046), a Tukey HSD comparison found only the mudstone and medium-grain sandstone were significantly different. Combining the two sandstone treatments and rerunning the ANOVA showed no significant effect (F_2,37_, p = 0.14).

**Fig 5:**
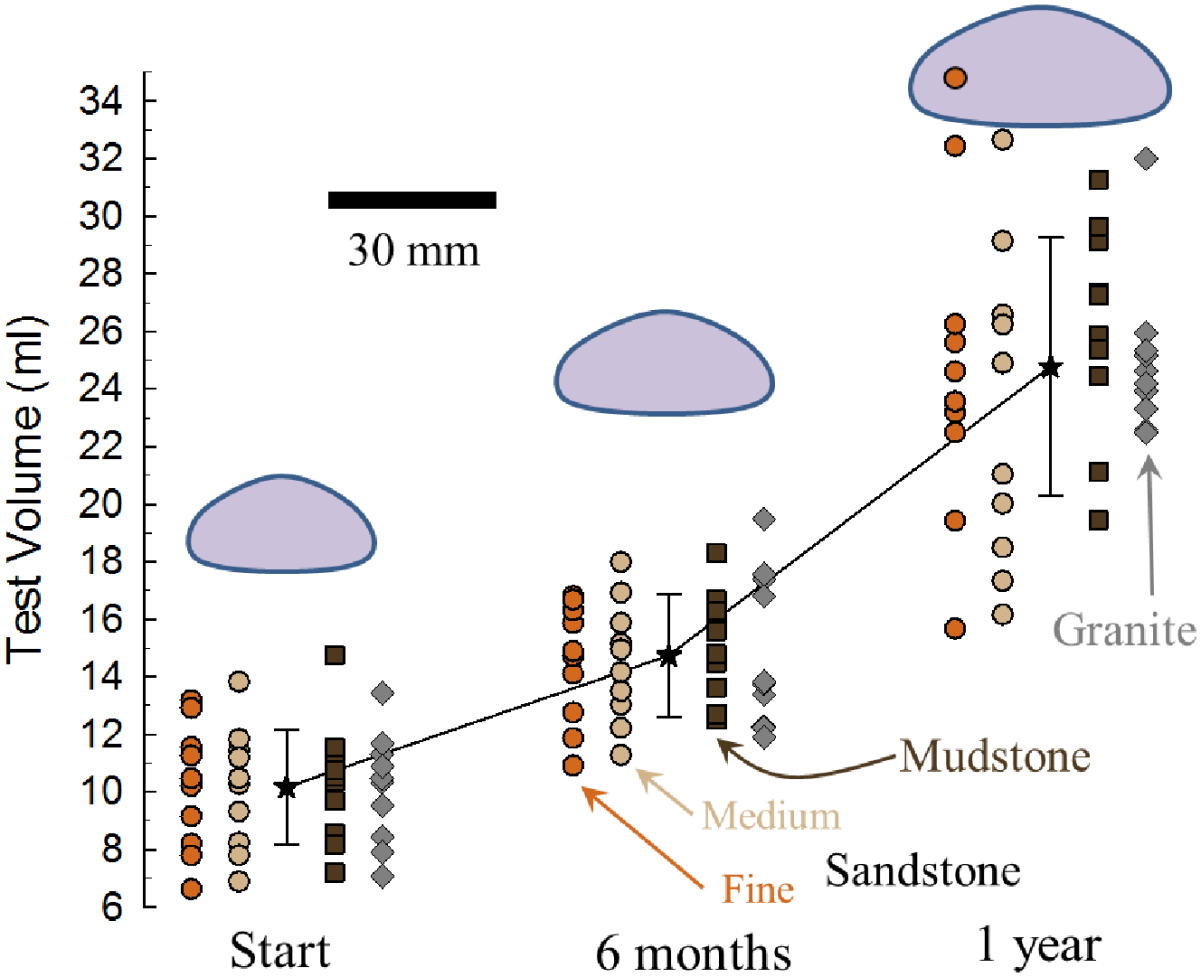
Sea urchin growth during one-year experiment. Test volume based on modified oblate spheroid estimate from test height and diameter [31]. Sandstone = circles (tan = medium-grain, orange = fine-grain); mudstone = brown squares; granite = gray diamonds (n = 10 each treatment). The black stars are the means (± sd) of all urchins at each time point. The urchin silhouettes are scaled-sizes of the average diameter and height for each of the three sampling time points (30 mm scale).

There was also no difference in overall growth rates among the four treatments (F_3,36_ = 0.47, p = 0.71) as measured in change of test volume over the year (Fig 5). However, there was significantly faster growth during the final six months versus the initial six months (Friedman χ^2^ = 28.9, p < .0001).

### Field measurements

**Fig 6:**
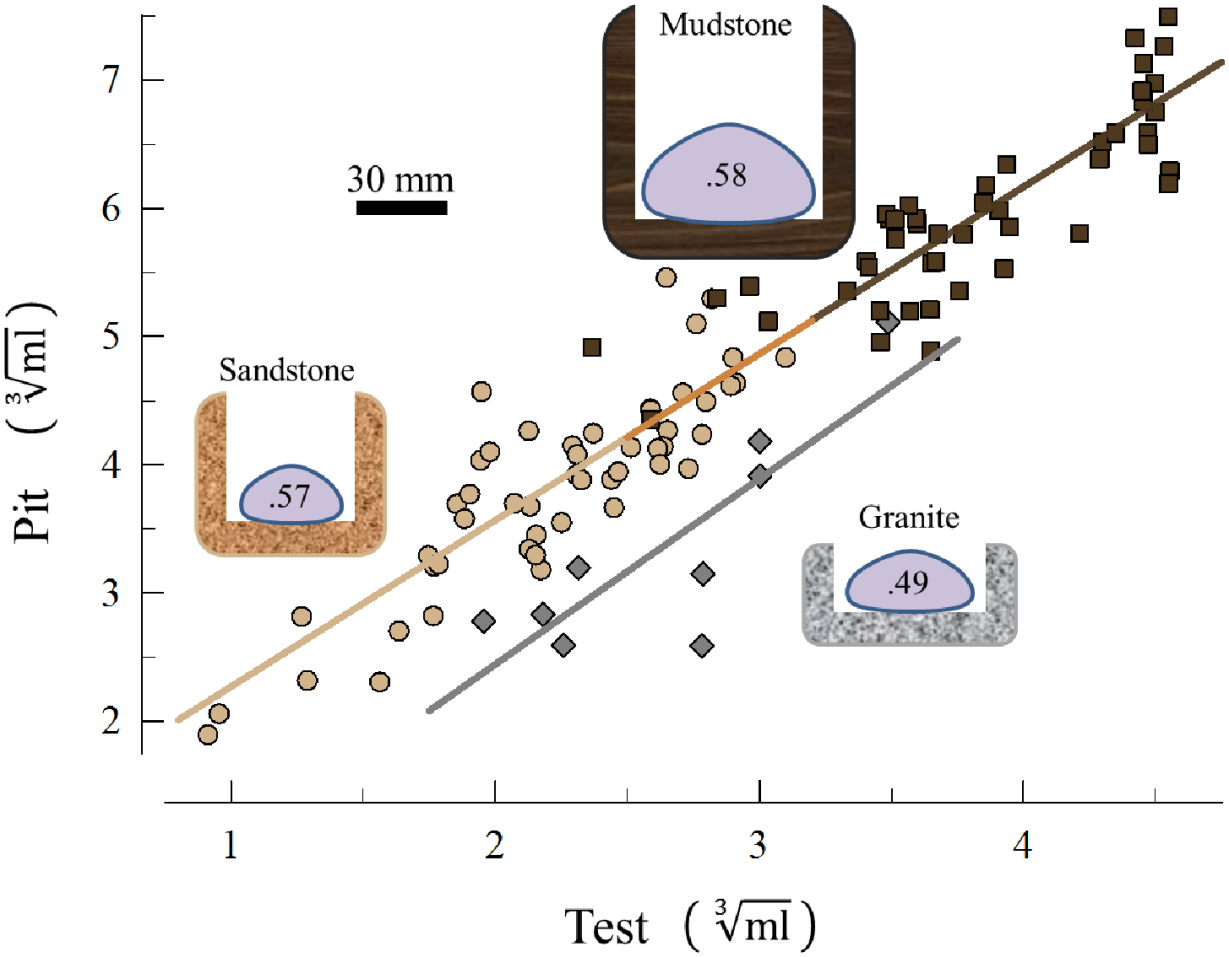
Field estimates of sizes of pits and urchin occupants. Cube-root of pit volume plotted against cube-root of test volume of urchin occupant. Sandstone = tan circles (n = 50); mudstone = brown squares (n = 47); granite = gray diamonds (n = 9). ANCOVA revealed no significant difference in slopes and the same regression described all three data sets (r^2^ = 0.91, F_1,95_ = 956.7, p<0.0001). Least squared means of sandstone and mudstone were not significantly different from each other but both were significantly different from granite. The silhouettes of urchins and pits are scaled-sizes of the average diameter and height of urchins, and depth and width of the pits. The urchin silhouettes contain the average height:diameter ratios of the tests, which had the same pattern (no difference between sandstone and mudstone, but both sedimentary rocks were different from granite).

As expected, the granite substrate had the shallowest urchin pits with the least volume for a given size of urchin occupant (Fig 6). There was no interaction in the ANCOVA and a common slope (1.33) accounted for the (cube-root) regression of pit volume as a function of urchin volume for the three sites (F_2,100_ = 1.68, p = 0.19). Although the mudstone pits were larger than the sandstone pits overall, this difference is attributable to the larger urchins at this site. The least square mean of the mudstone (4.91 ml^1/3^) was not significantly different from the sandstone pits (4.75 ml^1/3^; t = 1.23, p = 0.50) and a common regression describes the relationship of pit volume and urchin volume for both sedimentary substrata (Fig 6). The least square mean of both the mudstone and the sandstone pits were significantly different from the least square mean of the granite pits (3.79 ml^1/3^; t = 6.68, p < .0001 and t = 6.58, p < .000,1, respectively).

This pattern was also true for the shapes of the urchins as measured by the ratio of the height:diameter of the test. Mudstone and sandstone were not significantly different from each other but both were different from granite (F_2,103_ = 11.62, p < .0001).

### Waste collection experiment

**Fig 7:**
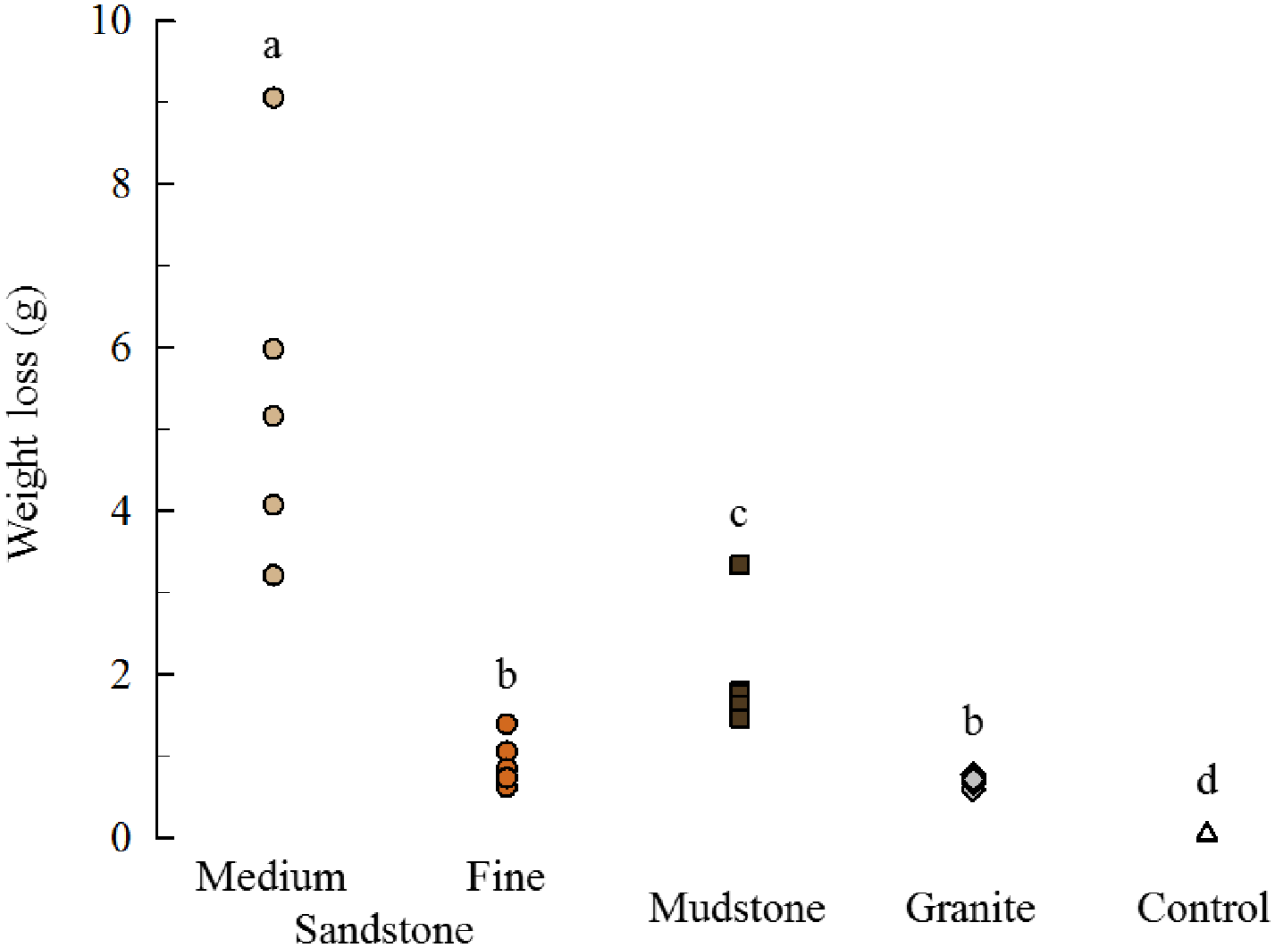
Dry weight change. The mass lost from each treatment was significantly greater than zero in all but the glass controls. The different letters above the data points indicate groups that are significantly different. Sandstone = circles (tan = medium-grain, orange = fine-grain); mudstone = brown squares; granite = gray diamonds; glass = open triangles (n = 5 each treatment).

In this short-term trial we did not observe obvious pit formation as we did in the one-year experiment. However, there were still significant reductions in the weight of all treatments except for the glass controls where the change was not different from zero (Fig 7, t = -.1274, p = 0.55). Similar to the one-year experiment, the medium-grain sandstone showed the highest levels of bioerosion and granite the least. Unlike the one-year experiment, the bioerosion in mudstone was significantly greater than the fine-grain sandstone and all the treatments except for granite and fine-grain sandstone were significantly different from each other (Fig 7, χ^2^ = 22.06, p < .001).

We calculated the inorganic percentage of the waste residue of the controls (45.7% ± 0.2) from the dry and ash weights. This percentage is the inorganic fraction due to fecal material alone (no rock substrate particles). We used this percentage and the dry and ash weights of the other treatments to calculate the additional inorganic content from bioerosion of the substrate. For example, the dry and ash weight residue of one of the medium-grain sandstone units was 2.94 g and 2.07 g, respectively. The inorganic mass lost from bioerosion for this unit was 2.07 –(45.7% x 2.94) = 0.72 g.

At the conclusion of the experiment we dissected all the urchins and measured the dry and ash weights of the contents of the digestive tracts. Similar to the waste residue from the buckets, the inorganic fraction of the gut contents in the controls (20.6% ± 2.5) was solely from the algae ingested (no bioerosion). We used this percentage and the dry and ash weights of the other treatments to calculate the additional inorganic content in the digestive tract from bioerosion of the substrate. For example, the dry and ash weight of the gut contents of one of the medium-grain sandstone units was 0.21 g and 0.075 g, respectively. The inorganic mass lost from bioerosion for this unit was 0.075 –(20.6% x 0.21) = 0.032 g.

The organic percentage of the different rock types were 1.6% ± 0.2 (medium-grain sandstone), 1.5% ± 0.1 (fine-grain sandstone), 6.3% ± 0.2 (mudstone), and 0.4% ± 0.1 (granite). The dry weights lost from each experimental unit were converted to inorganic mass lost using these values. For example, the dry-weight change in one medium-grain sandstone unit was 4.07 g and this was converted to 4.07 x (100%– 1.6%) = 4.00 g of inorganic mass lost.

**Fig 8:**
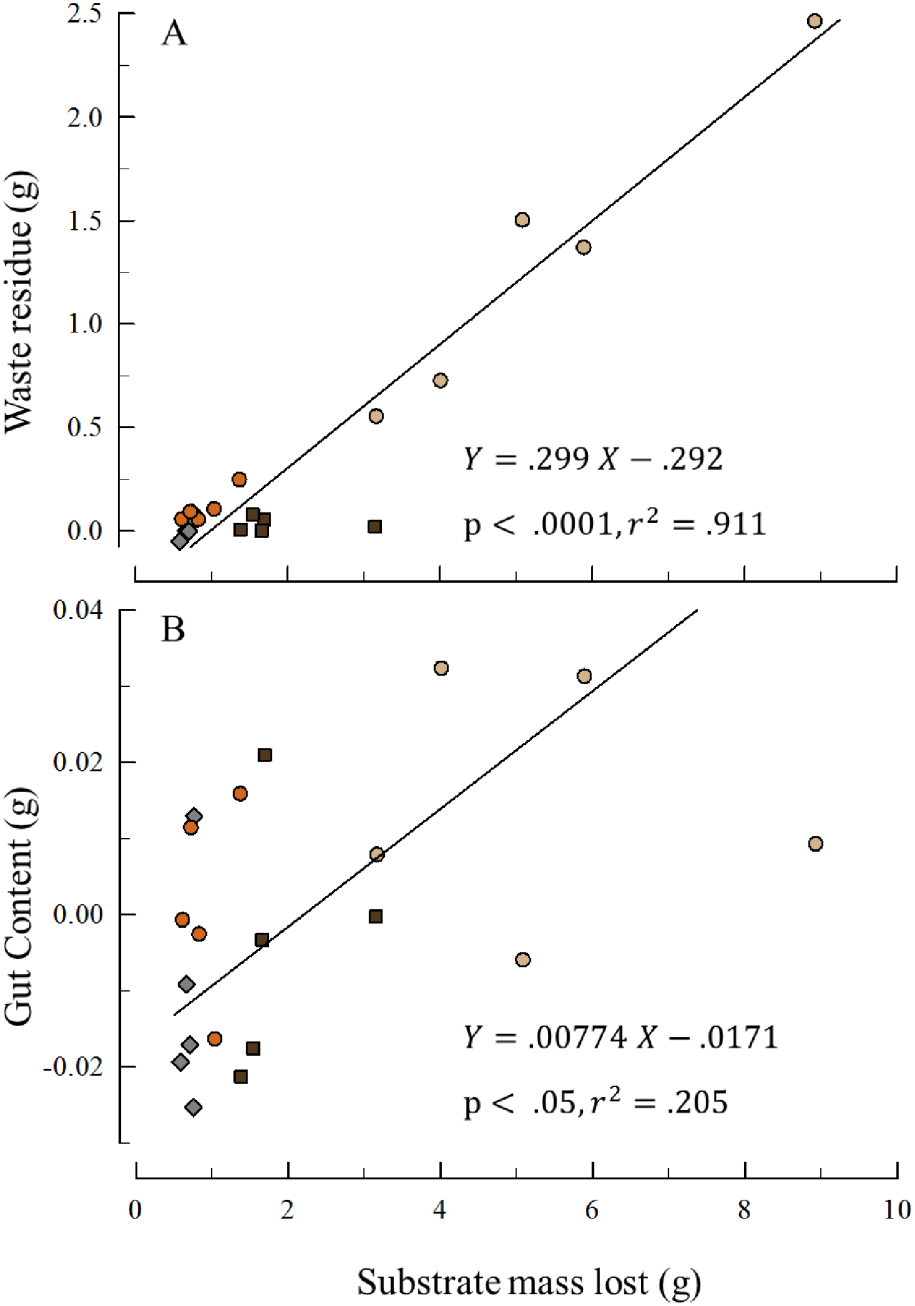
Inorganic weights from bioerosion. A. Waste residue from bioerosion. The inorganic weight after the fraction from fecal pellets subtracted. B. Gut Content from bioerosion. The inorganic weight after the fraction from ingested algae subtracted. In both plots sandstone = circles (tan = medium-grain, orange = fine-grain); mudstone = brown squares; granite = gray diamonds (n = 5 each treatment).

Fig 8 plots the inorganic waste residue and the inorganic gut content (both from bioerosion) as a function of the inorganic mass lost from each replicate. In both cases the slopes of these regressions are significant (residue: r^2^ = .91, t = 13.59, p < .0001; gut content: r^2^ = .21, t = 2.15, p < .05).

**Fig 9:**
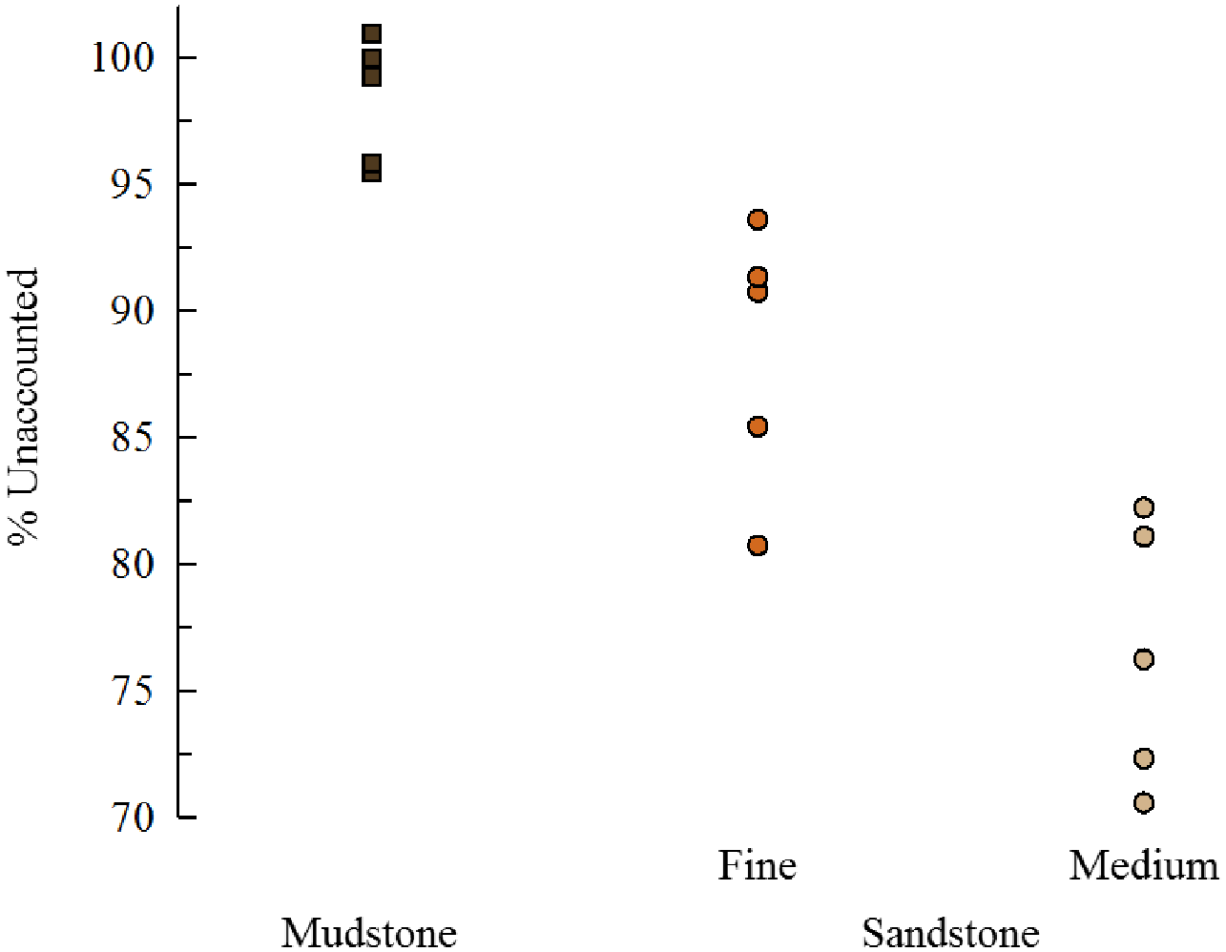
Unaccounted rock block change. Percent of the inorganic weight change in the rock block units not accounted for by the inorganic weight of the residue + inorganic gut content. All three groups are significantly different from each other (F_2,12_, p < .0001; Tukey HSD test). The rock with the smallest grain size (mudstone) has the highest percentage missing and medium-grain sandstone with the largest grain size the least percentage. Sandstone = circles (tan = medium-grain, orange = fine-grain); mudstone = brown squares (n = 5 each treatment).

For each replicate, the amount of inorganic substrate lost exceeds the sum of the waste residue and gut content. To test if grain-size explains this discrepancy, we analyzed if % Lost was associated with grain-size for the sedimentary treatments. Mudstone with the finest sedimentary particles size showed the most material unaccounted for and medium-grain sandstone with the largest particle sizes the least (Fig 9). This pattern is consistent with the hypothesis that the 100 micron sieve was too course to capture most of the material produced from bioerosion on the sedimentary units.

### Comparison of lab experiments

**Fig 10:**
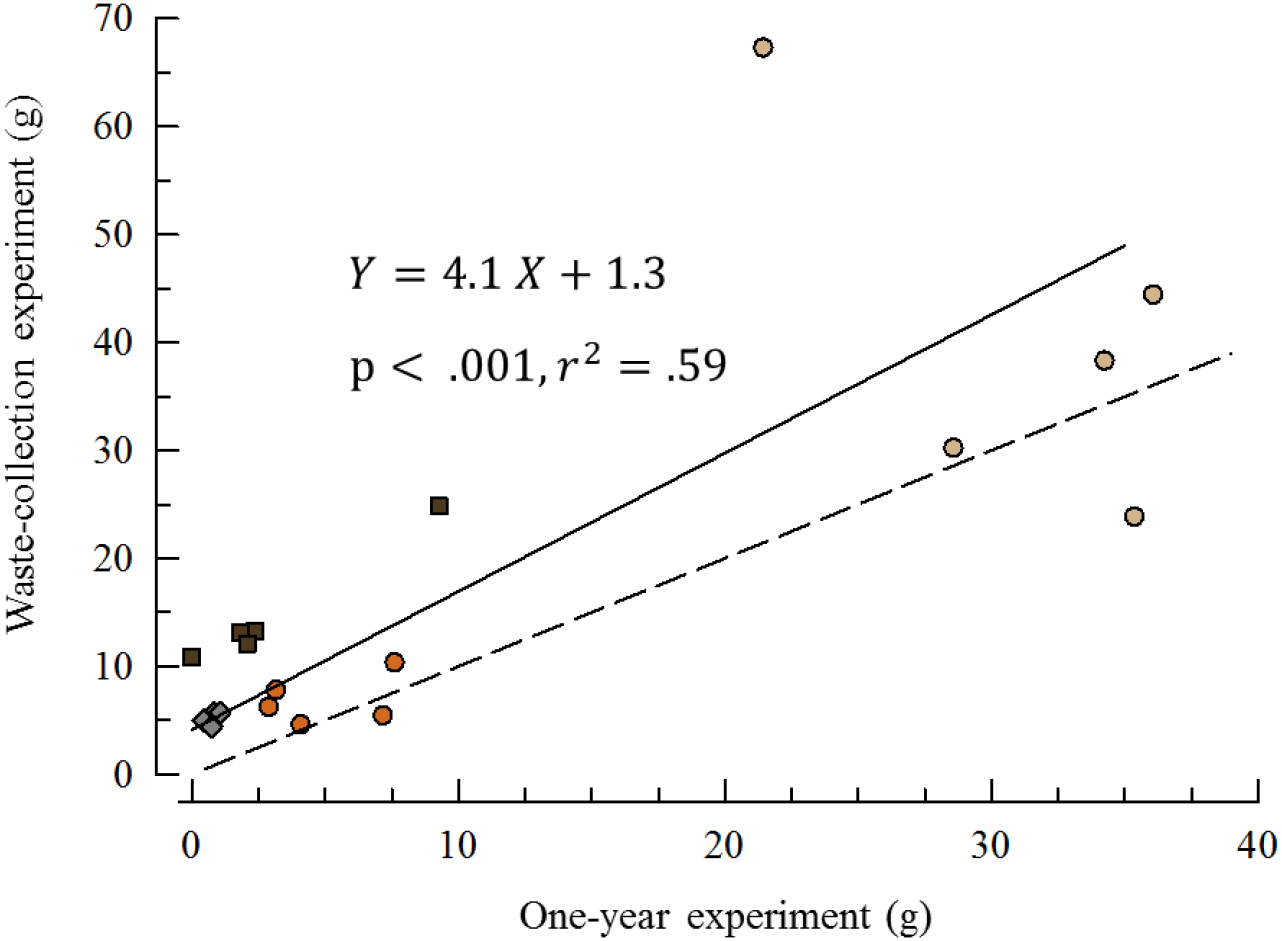
Bioerosion comparison between the two experiments. The rock block units were the same in each experiment so two estimates of bioerosion were derived for each. The values of the waste-collection experiment were prorated to the time frame of the one-year experiment (multiplied by 52/7). The dashed line (slope = 1) predicts the relationship if the two experiments yielded the same rates. The solid line fits the data with a Standard Major Axis (Model 2) regression. Both the slope and intercept of this regression show erosion rates were faster in the waste-collection experiment for all treatments. Sandstone = circles (tan = medium-grain, orange = fine-grain); mudstone = brown squares; granite = gray diamonds (n = 5 each treatment).

As expected we found a positive correlation in bioerosion estimates between the waste-collection and one-year experiments (Fig 10).

The bioerosion rates in the short-term waste collection experiment were much higher than the one-year experiment. The ratios of mean rates (waste-collection:one-year) were sandstone 1.5x; mudstone 5.3x; and granite 7.1.

## Discussion

This study demonstrated that sea urchins do in fact excavate pits in temperate reefs in rocks with different lithologies. Although it was logical to assume [16–18] pit-dwelling urchins were active in excavating these structures on temperate reefs, no previous studies provided experimental evidence of this process, or estimates of the resulting bioerosion rates. Previous studies have shown the biological and ecological effects of pits in rocky intertidal and temperate reefs on sea urchin growth, allometry, behavior [32], reproduction, physiology [33], predation avoidance [34], and on overall community structure [14]. In addition, urchins in pits are more difficult to dislodge than urchins on flat surfaces [35] enabling them to withstand the strong hydrodynamic forces of exposed rocky coasts.

While we have demonstrated that a single sea urchin has the ability to excavate a recognizable pit on sedimentary rock in less than one year (Fig 3), these lab studies have at least two limitations. First, we used urchins of the same size to standardize conditions across all four rock types to make bioerosion estimates comparable among treatments. We do not know if, or how, rates of bioerosion change with sea urchin size. Our measures on a mean-sized, per-urchin basis, were used to calculate overall bioerosion rates (see below). We do not think that a single urchin settles out of the plankton, excavates a pit as it grows, and remains there for the rest of its life. Temperate rocky shore sea urchins are “secondarily sessile” [36] but do move in response to a number of factors, including predators, food, and water-flow velocity [37–43]. The pit microhabitat is advantageous because it provides more surface area for tubefeet attachment, and spines can be used to wedge the organism inside, providing protection from waves and making it difficult for predators to dislodge them [34]. A single pit could be occupied by the same individual for months or years, but once that space becomes available, it is probably taken up by a new resident. Harder rock substrates like granite are less friable and pits on these substrata probably represent “legacy pits” and are the result of cumulative bioerosion [15].

The second limitation is that our study took place in the lab where we could not come close to duplicating the strong hydrodynamic forces of the intertidal. These forces may enhance the erosive grazing effects that urchins have on the substrate. Table 1 provides a baseline of bioerosion rates for urchins but suffers from the problems experienced with tooth loss (see below). The waste-collection experiment used the same experimental units and a fresh collection of urchins and produced much higher bioerosion rates. The bioerosion rates from the year-long study are conservative at best and likely underestimate the effect that urchins have in the field.

### One-year experiment

Although this study examined only three different types of sedimentary rocks, our results suggest there may be a gradient of bioerosion rate associated with grain size. However, other sedimentary rock properties such as mineralogy, cementation, and degree of compaction undoubtedly influence both bioerosion as well as physical erosion rates.

We were surprised that the “softer” mudstone showed lower rates of bioerosion than sandstone because the mudstone surface is easily etched with a pocket knife. In addition, at Palomarin we found mudstone cobbles washed ashore with bore holes that contained the remains of the clams that made them (shells of a species in the family Pholadidae, S2 Fig). The rock saw we used to prepare the units shattered the mudstone when the rotating blade touched the rock. Although the disc sander produced a smooth surface on the mudstone, it was not water-cooled and during the process heated the units. We do not know if this heating altered the physical properties of the surface that the urchins interacted with or whether the rates of bioerosion were altered. To mitigate this potential confounding factor, we scraped the surfaces of the mudstone (and all other units, except the smooth glass controls) with a serrated knife when preparing them for the waste-collection experiment (see below).

There was a clear difference in the growth rates of all urchins in the four treatments between the first and second halves of the experiment. During the first six months, the mean volume of all urchins increased 45%, whereas during the final six months they increased 68%. Some of this difference may be associated with feeding on the fresh-frozen kelp during the first six months and the rehydrated kelp the remaining six months. However, we attribute much of the growth difference to the high PO_4_^-3^ (and low Ca and Mg) levels associated with the way we initially handled the rehydrated kelp. The resulting tooth loss during the first six months undoubtedly affected feeding efficacy and the reallocation of resources to repair teeth most likely contributed to the overall lower growth rates. In addition, because the urchins were not rasping the substrate with a full complement of teeth, the bioerosion rates derived from this experiment are likely underestimates of their abilities in the field.

### Field measurements

Ideally one would select field sites that had healthy populations of sea urchins in pits. Historically, the Bodega Bay site had high-density populations of intertidal purple sea urchins and for other studies we sampled there in 1985 [44] and re-sampled in 2007–2009 [45]. However, in August of 2011, a geographically localized (100 km of coastline) mass-mortality event functionally extirpated both *S*. *purpuratus* and a sea star (*Leptasterias sp*.) from this site [29]. There were a limited number of accessible coastal sites outside of the mass mortality zone with the same type of granite as Bodega Bay [23]. The closest granite site was McClure’s Beach (38° 11ʹ 28.92ʺ N 122° 57ʹ 57.22ʺ W), which is immediately adjacent to the reported mass mortality zone. However, our efforts to find living urchins at this site were unsuccessful. The next closest site with this type of granite, Montara Lighthouse (37° 32ʹ 14.46ʺ N 122° 31ʹ 11.13ʺ W), was extremely difficult to access because of the craggy and dangerous terrain along this part of the coast. We managed to locate one tidepool at this site that had 39 sea urchins, 9 of which were in pits that we could access to measure.

Urchin pits (or “burrows”) whether occupied or unoccupied are easily identified, distinctive features in the rocky substratum (Fig 1, also in [29] their Figure 3, and in [15] their Figure 1). They are so distinctive that Jurgens et al. [29] used counts of these structures to estimate pre-mass mortality urchin populations at some sites. We selected 14 (unoccupied) pits at Bodega Bay that were not obviously overgrown by encrusting algae, measured their dimensions, and compared them to the size of the occupied pits at Montara Lighthouse. Because there was no significant difference, we are confident that our estimates of rates of bioerosion and pit formation of the granite substratum from Bodega Bay are sound. In addition, the ANCOVA showed there was not a significant interaction between pit size and urchin size across all three rock substrates (a common slope describes the relationship).

Between the two types of sedimentary rocks, there was little overlap in either urchin volume (20.0% of the range) or pit volume (19.8% of the range), and yet a common regression (slope and intercept) provided a very good fit to the relationship. It would be instructive to see if this relationship holds for other sedimentary sites along the distribution of *S*. *purpuratus*.

The disparity in the time it would take a single urchin to make a site-specific average size pit between the sandstone (4.5 and 31.9 yr for medium-grain and fine-grain, respectively) and mudstone (143.2 yr) is due primarily to the different sizes of pits (and urchins making them). Using the rock density and lab-based bioerosion estimate of mudstone, it would take a single urchin only 41.3 yr to make a pit at Palomarin (mudstone site) the size of the average-size pit at Bean Hollow (the sandstone site). What seems to be driving the disparity is the difference in sizes of the sea urchins at the two sites (Fig 6).

### Waste collection experiment

The glass plates served as satisfactory controls because the change in weight was not significantly different from zero indicating that both the waste collected during the experiment, and the gut content at the end, were solely from the fecal material, and algae ingested, respectively. In the one-year experiment the fine sandstone showed more bioerosion than the mudstone, but in the short-term experiment the opposite was true. The difference indicates that the preparation of the mudstone units for the one-year experiment (disc sanding) may have inadvertently altered the physical properties of the mudstone and affected the ability of the urchins to erode the surfaces. Slowly scraping the surfaces of these units with a serrated knife (preparation for short-term experiment) seems to have eliminated this issue. From our visual and tactile inspection of the mudstone units we could not perceive any difference in the surface texture of the units after disc sanding, but it is clear that the relative rates of bioerosion were affected.

Our observations of both fecal pellets infused with sedimentary particles (S3 Fig), and boluses composed of sedimentary particles in the digestive tracts (S1 Fig), showed that one mechanism of purple sea urchin bioerosion is substrate ingestion. However, it was not possible to assign all of the waste material collected in the buckets to substrate ingestion because some of it could have come from the scraping of spines against the rocks (Fig 8A). The positive correlation between inorganic gut content and the inorganic material lost from the rock substrates (Fig 8B) confirms our earlier observations that substrate ingestion accounts for some fraction of the bioerosion and the waste residue in the buckets.

The two week difference between how long the urchins spent on the units (7 weeks) and the collection of waste material (5 weeks) does not account for the large difference between the inorganic material lost from the blocks, and the inorganic residue plus inorganic gut content (adjusted for glass controls, Fig 8). During the experiment, we periodically observed intact fecal pellets composed primarily of sedimentary particles clustered on the aboral surface around the anus (S3 Fig). These pellets were delicate (unlike fecal pellets produced from algae), difficult to manipulate, and usually broke apart when disturbed. Many attempts to isolate them for photographs were unsuccessful (but see S3 Fig). When these pellets dispersed, many of the sedimentary particles appeared to be too fine to be captured in the residue collected on the 100-micron sieve and much of this material was probably rinsed away in the discarded filtrate.

### Comparison of lab experiments

We attribute the higher bioerosion estimates in the waste collection experiment to the problem of tooth breakage/loss in the early stages of the one-year experiment. This observation supports the hypothesis that teeth (rather than spines) are the primary source of bioerosion. It also indicates that our estimates of bioerosion from the one-year experiment are conservative.

## Conclusion and broader implications

In previous studies we surveyed urchins in rock pools from the three sites (S1 Table). Density estimates from these rock pools (n = 10) range from 33 m^-2^ to over 1000 m^-2^ with a mean value across all pools and sites of 368 m^-2^. At the sandstone site (Bean Hollow) the density was 615 m^-2^ and the estimates of bioerosion were 199.1 t yr^-1^ ha^-1^ for medium-grain and 29.4 t yr^-1^ ha^-1^ for fine-grain sandstone. The mean across all rock types was 58.9 t yr^-1^ ha^-1^. For comparison with the waste-collection rates these estimates were 250.9 t yr^-1^ ha^-1^ for medium-grain and 42.3 t yr^-1^ ha^-1^ for fine-grain sandstone, and 83.4 t yr^-1^ ha^-1^ across all rock types.

Most research on bioerosion and sediment production focuses on coral reef ecosystems and the activities of parrotfish (Scaridae) and tropical sea urchins (Echinometridae and Diademidae) eroding biogenic carbonate substrata. The highest rates of bioerosion in these systems are on the same order of magnitude reported here. For the parrotfish *Sparisoma viride* [46] in Bonaire, and *Chlorurus gibbus* [47] on the Great Barrier Reef, estimates were 54 and 55 t yr^-1^ ha^-1^. Estimates of rates for the tropical sea urchins *Diadema antillarum* [48] in Barbados, and *Echinometra mathaei* [49] in St. Croix, were 53 and 39 t yr^-1^ ha^-1^, respectively. There are two important differences between these tropical data and the bioerosion estimates in this study. First, the substratum in the tropical studies was coral “rock”, or carbonate from a living reef. The sedimentary and metamorphic substrata used in this study are tougher and more difficult to abrade [50], yet the urchins showed significant erosion of even the granite treatment. Second, the tropical studies were short-term and several reasonable, yet key assumptions were made to estimate bioerosion, e.g., feeding rates and turnover-time of material in the gut (~ 10x d^-1^ for parrotfish and ~ 1x d^-1^ for urchins). In contrast, the bioerosion estimates reported here for *S*. *purpuratus* are simply differences in the weight of the substrates with and without sea urchins over the course of one-year in the long-term experiment, and 7 weeks in the waste-collection experiment.

What is the total amount of sediment produced by purple urchins and how does it compare to other sources? This species ranges from the intertidal to depths of 90 m [51] and latitudinally from southeast Alaska (58° N) to Baja California (28° N) [52]. This broad geographic range includes approximately 2.1 x 10^3^ km of habitat [53] and 58 rivers [54]. These rivers transport sediment to the coast and have a median sediment load of 1.3x10^6^ t yr^-1^ [54]. The conservative per urchin bioerosion rate (Table 1) for sandstone is 18.6 g yr^-1^, and across all four rock types is 10.2 g yr^-1^ (these values increase to 23.9 g yr^-1^ and 16.9 g yr^-1^, respectively, for the waste-collection experiment). Using both the conservative and waste-collection experiment estimates to calculate how many urchins are required to produce as much sediment as a median-size river yields 55–69 (sandstone) to 77–127 (mean for all four rock types) billion urchins. It is difficult to estimate the total number throughout the range. However, using the density estimates (S1 Table) and geographic range indicates that between 0.7 and 1.7 hectares of urchins per km of coastline would equal the median sediment load of a river. Even if the purple sea urchin population is within an order of magnitude of the 55–127 billion mark, our estimates show they are a significant source of coastal sediment.

Historically, sea otters (*Enhydra lutris*) played a keystone role in this system and have a top-down effect on sea urchins and kelp [55–57]. When otters are present, their predation keeps urchin populations low, which in turn reduces urchin grazing allowing kelp to thrive. Without sea otters, urchin populations increase so dramatically their grazing eliminates kelp and most other macroalgae resulting in urchin barrens. In these habitats, it is likely the urchins are rasping the rock surface even more intensely and bioeroding at even higher rates because there is little-to-no macroalgae and one of the few available food resources is the biofilm on the substrate. Because of their effect on urchin populations, the presence/absence of sea otters is not only determining the states of the kelp/barrens communities, it is likely altering the spatial and temporal rates of bioerosion and sediment production. There are several “knock-on effects” of the otter-kelp trophic cascade that impact a variety of processes including food web dynamics and atmospheric carbon sequestration [58]. The urchin-mediated knock-on effect of bioerosion is a potentially important variable in sediment production and temperate reef coastal erosion that deserves further investigation.

Finally, our study was limited to one metamorphic and three sedimentary rock types which represent only a fraction of the substrata of nearshore temperate reefs. Despite this limited sampling, the two types of sandstone (from Bean Hollow) showed disparate rates of bioerosion suggesting the potential variation that probably exists in the field both between and within sites. Although these estimates from sandstone were variable, the rates of bioerosion were consistent between the two experiments indicating that the new techniques employed in this study to measure bioerosion are valid. Another limitation of this study is the laboratory setting—on the one hand we were able to isolate the effects of sea urchin activity, but these estimates came at the expense of quantifying bioerosion in a natural setting. A number of factors in the field including high-energy waves likely enhance the bioerosive effects of grazing urchins. A logical next step in this line of inquiry is modifying and applying these techniques to field-based studies at several sites with a wider range of rock types.

## Supporting information

S1 Fig Photographs of urchin dissection and waste-collection experimental setup. A. Dissection of one of the urchins from the medium-grain sandstone treatment at the end of the year-long experiment (test diameter = 46 mm) revealing boluses of pellets comprised of sandstone particles in the digestive tract (teased open). B. Close up of pellets. C. System designed to capture wasted from the urchins bioeroding the rock on the units enclosed in the cages. The plastic buckets the units are mounted in are 4.7 L.
(TIF)

S2 Fig Photograph of Palomarin mudstone with pholad bore holes. The arrows indicate the articulated shells of the clams. The diameter of the coin is 21 mm.
(TIF)

S3 Fig Photographs of fecal pellets composed of sedimentary particles. Unlike fecal pellets produced from urchins feeding on algae, these sedimentary-particle pellets were delicate and usually broke apart when touched. A. Medium-grain sandstone pellet. B. Mudstone pellet. C. Medium-grain sandstone treatment with disarticulated sedimentary fecal pellets surrounding the anus. Diameter of sea urchin = 36 mm.
(TIF)

S1 Table Tidepool dimensions, sea urchin counts, densities, and sizes from the three different sites. All sea urchins were removed from the tidepool and test diameters (Diam) measured with knife-edge vernier calipers. Pools sampled between July 2, 2007 and June 26, 2009.
(PDF)
