# Supplementary figures and images for: Bioerosion by pit-forming, temperate-reef sea urchins: History, rates and broader implications
Source: PLoS One. 2018 Feb 21;13(2):e0191278. doi: 10.1371/journal.pone.0191278 (PMC5821313; doi:10.1371/journal.pone.0191278)

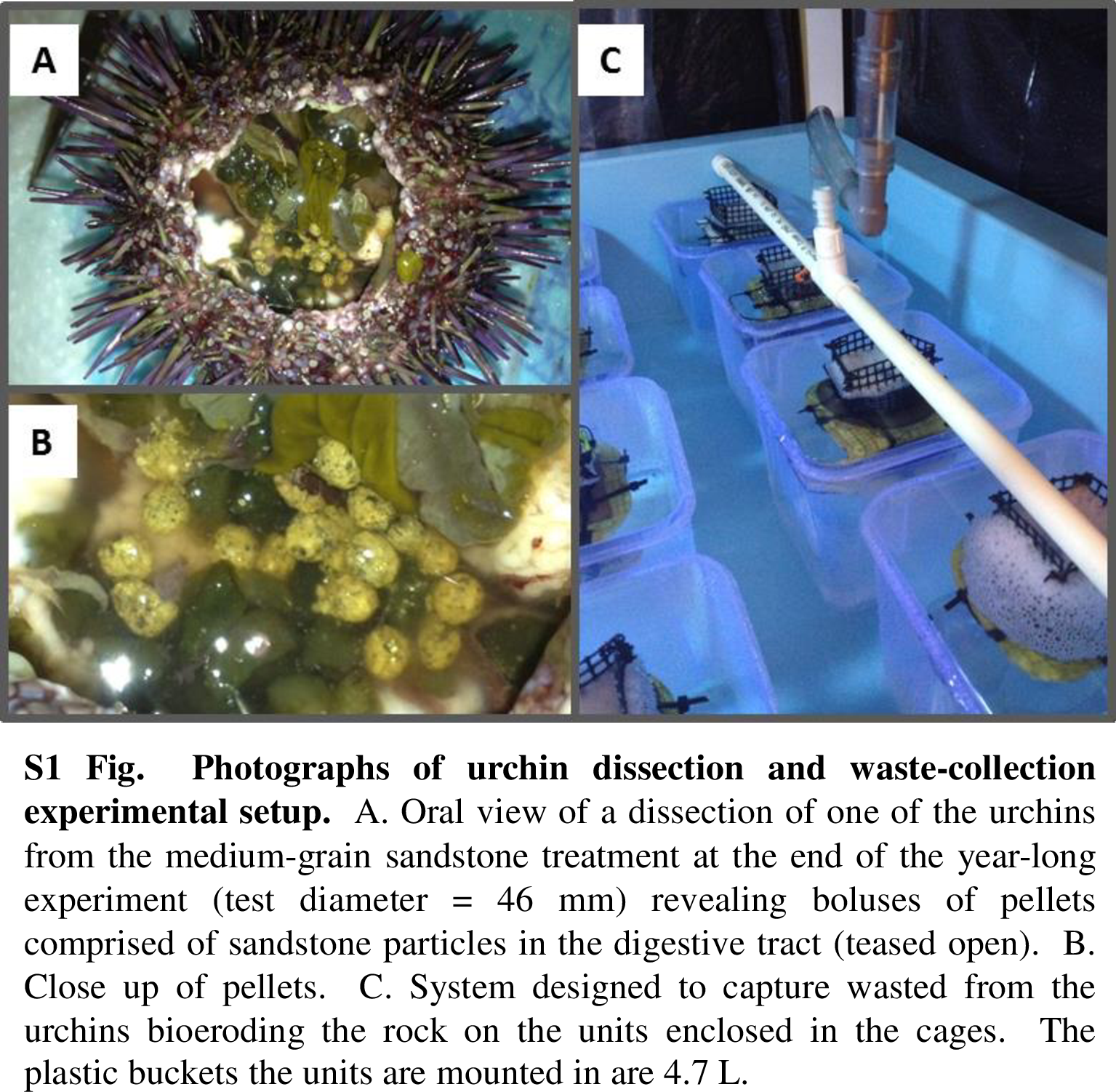

Supplement: S1 Fig — A. Dissection of one of the urchins from the medium-grain sandstone treatment at the end of the year-long experiment (test diameter = 46 mm) revealing boluses of pellets comprised of sandstone particles in the digestive tract (teased open). B. Close up of pellets. C. System designed to capture wasted from the urchins bioeroding the rock on the units enclosed in the cages. The plastic buckets the units are mounted in are 4.7 L. (TIF) [file pone.0191278.s001.tif]

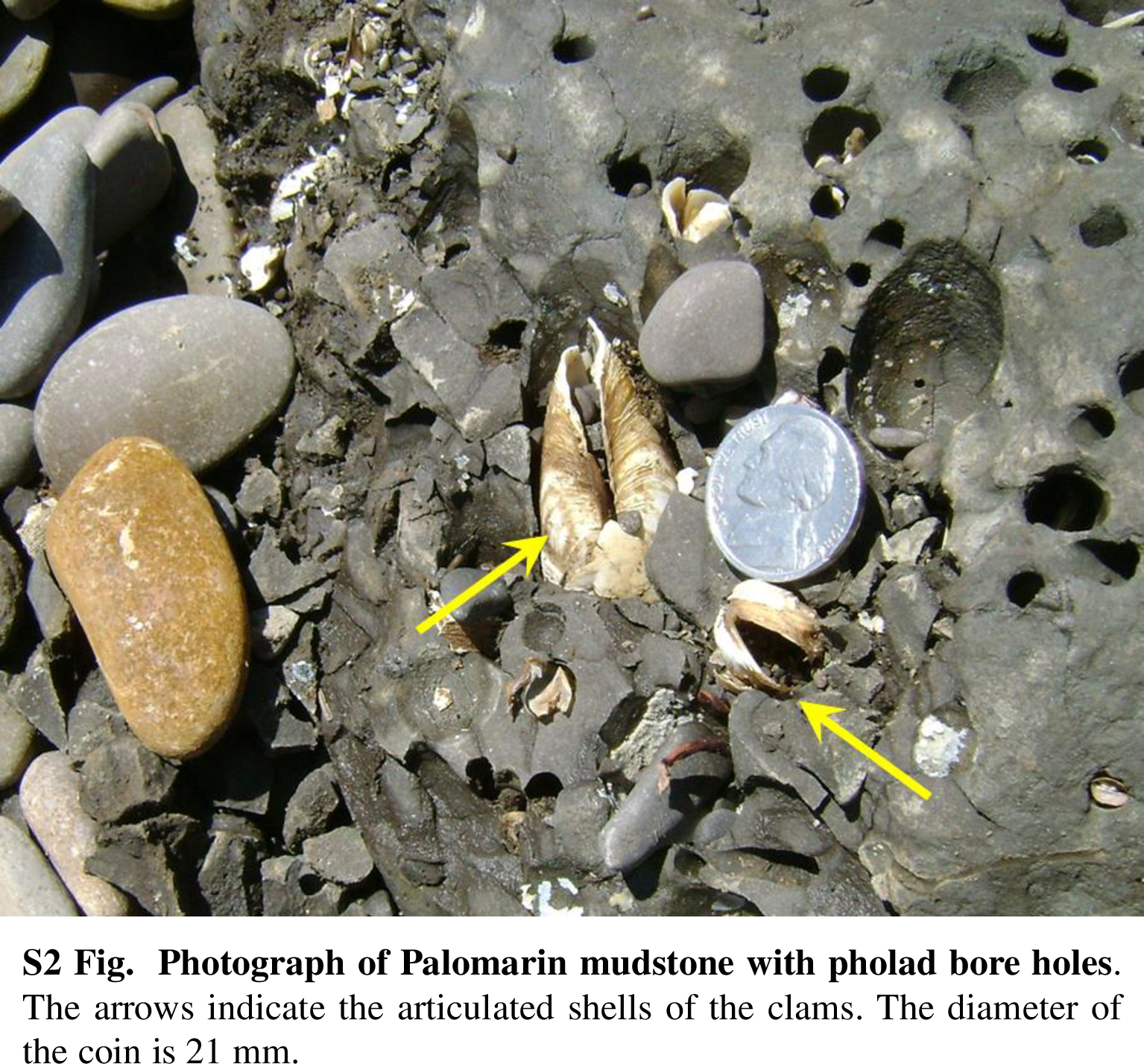

Supplement: S2 Fig — The arrows indicate the articulated shells of the clams. The diameter of the coin is 21 mm. (TIF) [file pone.0191278.s002.tif]

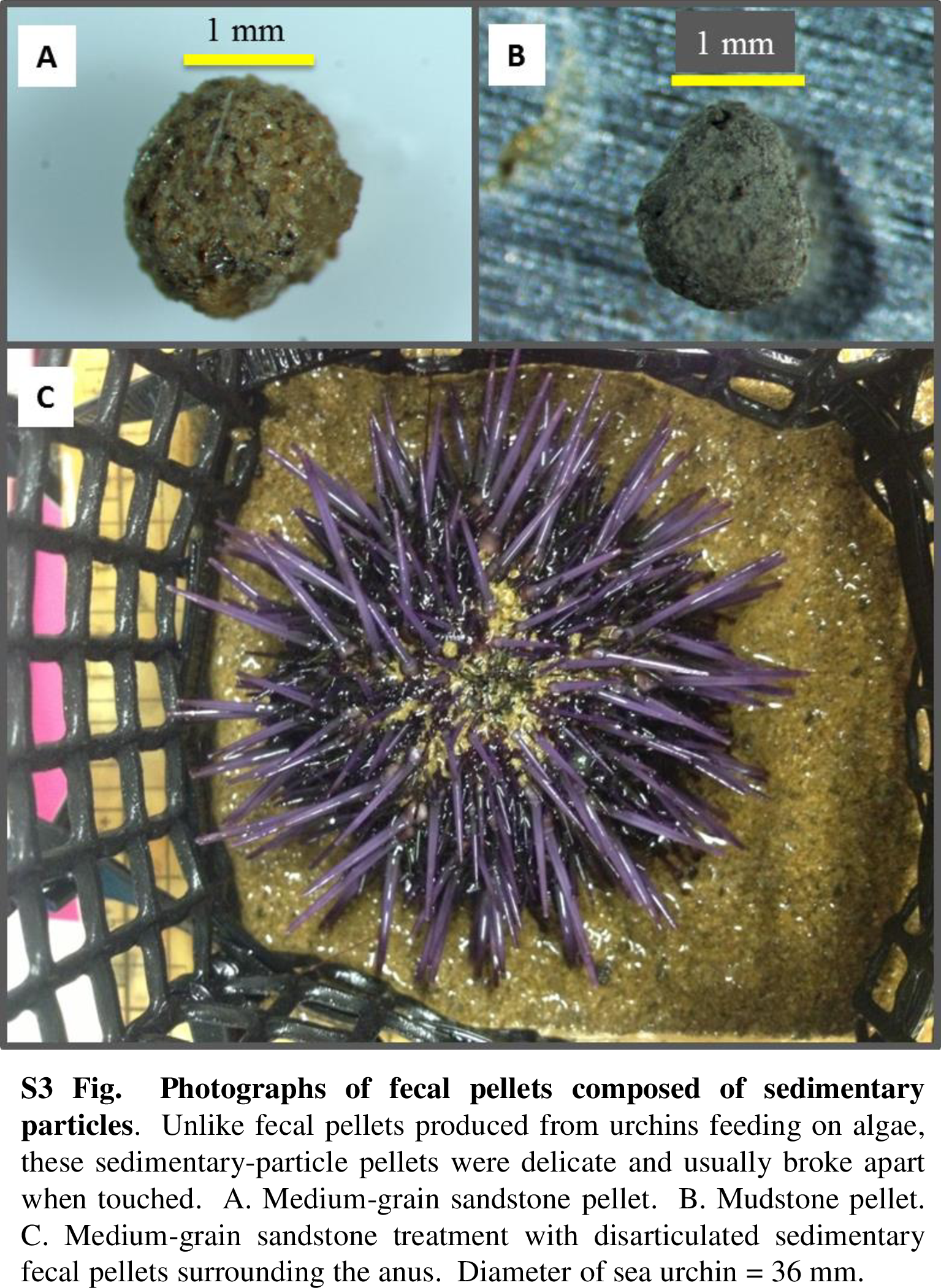

Supplement: S3 Fig — Unlike fecal pellets produced from urchins feeding on algae, these sedimentary-particle pellets were delicate and usually broke apart when touched. A. Medium-grain sandstone pellet. B. Mudstone pellet. C. Medium-grain sandstone treatment with disarticulated sedimentary fecal pellets surrounding the anus. Diameter of sea urchin = 36 mm. (TIF) [file pone.0191278.s003.tif]
