# Supplementary material for: Bioerosion by pit-forming, temperate-reef sea urchins: History, rates and broader implications
Source: PLoS One. 2018 Feb 21;13(2):e0191278. doi: 10.1371/journal.pone.0191278 (PMC5821313; doi:10.1371/journal.pone.0191278)
Supplement: S1 Table — All sea urchins were removed from the tidepool and test diameters (Diam) measured with knife-edge vernier calipers. Pools sampled between July 2, 2007 and June 26, 2009. (PDF) [file pone.0191278.s004.pdf]

S1 Table. Tidepool dimensions, sea urchin counts, densities, and sizes from the three different sites. All sea urchins were removed from the tidepool and test diameters (Diam) measured with knife-edge vernier calipers. Pools sampled between July 2, 2007 and June 26, 2009.

Site	Pool (m)		Sea urchins		
Bodega (granite)	Length	Width	N	# m^{-2}	Diam (mm)
1	0.35	0.60	180	857.1	28.10 ± 10.72
2	4.50	0.60	245	90.7	32.01 ± 11.43
3	3.50	1.75	200	32.7	19.30 ± 14.00
4	2.30	0.80	649	352.7	19.65 ± 7.02
Palomarin (mudstone)					
1	1.25	0.90	323	287.1	43.64 ± 12.91
2	2.10	0.80	197	117.3	42.93 ± 15.11
3	2.85	0.65	111	59.9	49.79 ± 10.99
Bean Hollow (sandstone)					
1	0.55	0.48	266	1007.6	21.17 ± 11.14
2	1.05	0.72	146	193.1	35.72 ± 13.22
3	0.75	0.68	328	643.1	24.68 ± 15.20
